# Association between fruits and vegetables intake and frequency of breakfast and snacks consumption: a cross-sectional study

**DOI:** 10.1186/1475-2891-12-123

**Published:** 2013-08-27

**Authors:** Giacomo Lazzeri, Andrea Pammolli, Elena Azzolini, Rita Simi, Veronica Meoni, Daniel Rudolph de Wet, Mariano Vincenzo Giacchi

**Affiliations:** 1 Department of Molecular Developmental Medicine, University of Siena, Via A. Moro 2, Siena 53100, Italy; 2 CREPS – Centre of Research for Health Education and Promotion, University of Siena, Siena, Italy; 3 Local Public Health Unit 7, Siena 53100, Italy; 4 Department of Biomedical and Neuromotor Sciences, University of Bologna, Bologna, Italy

## Abstract

**Background:**

There are very few studies on the frequency of breakfast and snack consumption and its relation to fruit and vegetable intake. This study aims to fill that gap by exploring the relation between irregular breakfast habits and snack consumption and fruit and vegetable intake in Tuscan adolescents. Separate analyses were conducted with an emphasis on the potentially modifying factors of sex and age.

**Methods:**

Data was obtained from the 2010 Tuscan sample of the Health Behaviour in School-aged Children (HBSC) study. The HBSC study is a cross-sectional survey of 11-, 13- and 15-year-old students (n = 3291), selected from a random sample of schools. Multivariate logistic regression was used for analyzing the food-frequency questionnaire.

**Results:**

A significant relation was found between low fruit and vegetable intake and irregular breakfast habits. Similarly, low fruit intake was associated with irregular snack consumption, whereas vegetable intake did not prove to be directly related to irregular snack consumption. Different patterns emerged when gender and age were considered as modifying factors in the analyses. A statistically significant relation emerged only among female students for irregular breakfast habits and fruit and vegetable intake. Generally, older female participants with irregular breakfast habits demonstrated a higher risk of low fruit and vegetable intake. Age pattern varied between genders, and between fruit and vegetable consumption.

**Conclusions:**

Results suggest that for those adolescents who have an irregular consumption of breakfast and snacks, fruit intake occurs with a lower frequency. Lower vegetable consumption was associated with irregular breakfast consumption. Gender and age were shown to be moderators and this indicated the importance of analyzing fruit and vegetable intake and meal types separately.

This study also confirmed that health-promotion campaigns that aim to promote regular meal consumption and consumption of fruits and vegetables need to take into account gender and age differences in designing promotional strategies. Future research should identify evidence-based interventions to facilitate the achievement of the Italian guidelines for a healthy diet for fruit, vegetables and meals intake.

## Background

From a public health point of view, a high consumption of fruits and vegetables reduces the risk of heart disease and some forms of tumor [1-5]. Furthermore, eating enough fruits and vegetables during childhood and adolescence is important for at least three reasons. First, as childhood and adolescence are phases of growth, the body requires more nutrients [6,7]. Second, the eating patterns established during childhood and adolescence tend to continue in adulthood [8]. Third, childhood and adolescence are key phases for easily modifying eating habits, as opposed to adulthood when such habits tend to be more rigid [9]. In Italy, the main national-level study that investigated fruit and vegetable intake of adolescents is the Italian HBSC survey [10]. The percentages of adolescents who report eating fruits and vegetables with a daily consumption (“Once every day” and “Several times every day”) are very low. The daily fruit consumption prevalence was found to be 45.5% for 11-year-olds, 39.9% for 13-year-olds and 38.4% for 15-year-olds, while the daily vegetable consumption prevalence was 21.1%, 19.6%, 20.2% respectively [11].

The various factors determining fruit and vegetable consumption among adolescents include frequency of meal consumption [12]. Although this factor was not studied in depth, the consumption of fruits and vegetables seems to be higher among children with regular meal habits [13]. Less healthy food choices are made by adolescents who miss breakfast at least once a week compared with those who have breakfast every day. It has also been noted that there is a higher frequency of snack consumption in both healthy as well as unhealthy dietary behaviour and lifestyle patterns [14]. Large quantities of soft drinks, white bread and sweets are consumed by those who tend to skip meals. Moreover, this group also tends to consume less fruits and vegetables [15]. Adolescents who consume meals frequently have a higher intake of fruits and vegetables. On the contrary, teenagers who consume meals less frequently tend to eat less fruits and vegetables [16]. Other studies showed that children who miss at least one main meal per day eat less fruits and vegetables compared with those who do not skip any meals. Age was not a determining factor in this analysis [17,18]. A study on 13–15 year-old school children, carried out by Lien et al., produced a meal-score system by combining the weekly frequency of breakfast consumption. Higher scores were associated with a higher consumption of fruits and vegetables (combined amount) [19]. However, this association did not emerge in other studies [20,21]. As a result, it can be concluded that there is still no established association between meal frequencies and fruit and vegetable intake among adolescents. No earlier study on this association has been conducted in our country and there is a lack of consistent evidence even in international studies.

The aim of this research is to observe whether there is any direct association between the frequency of breakfast and snack consumption and fruit and vegetable intake among 11-, 13- and 15-year-old Tuscan students. Moreover, this study investigates the potentially modifying effect of sex and age. The variables in this research such as fruit and vegetable intake and frequency of snack and breakfast consumption were observed separately because associations may vary by fruits and vegetables and by meal type.

## Methods

### Study design and population

Data was taken from the Tuscan 2010 “Health Behaviour in School-aged Children (HBSC) study’, a WHO cross-national survey. The methods employed in gathering this data are described in detail elsewhere [10]. Parents had to give their consent for their children to be part of the survey. The Ethics Committee of the National Institute of Health, which approved the protocol, agreed to the use of an opt-out consent form, which implied that a child will be included by default unless his or her parents chose to “opt-out” by explicitly refusing consent. Data collection is anonymous and the demographic information collected (gender, year and month of birth, nationality, nationality of parents) does not permit identification of the individual student. Data is collected every fourth year on children aged 11, 13 and 15 out of a random sample of schools (also known as cluster sampling).

The HBSC study uses an anonymous self-administered questionnaire, which was according to international standards and distributed in schools [22]. All member countries are involved in a continuous process of development and validation of the questionnaire. There are several studies on many topics that have demonstrated the validity of the questionnaire [23-31].

### Measurements

#### Dependent variables

The food frequency questionnaire (FFQ), a module in the HBSC questionnaire, was used to measure both fruit and vegetable consumption. The question, “How many times a week do you usually eat/drink…” was followed by a list of food and beverage items, including “Fruits” and “Vegetables”. The options were: “Never”, “Less than once a week”, “Once a week”, “2-4 days a week”, “5-6 days a week”, “Once every day” and “Several times every day”. The variables were dichotomized to define low fruit and vegetable consumption as 5–6 days per week or less. The Italian Guidelines for a Healthy Diet (IGHD) recommends 5 or more portions of fruits and vegetables daily [32].

#### Independent variables

The various frequencies of breakfast and snack consumption were considered independent variables. To collect data relative to these variables, students were asked to respond to two questions: “On weekdays: How often do you usually have breakfast (more than a glass of milk, cup of tea or fruit juice)?” and “How often do you usually have snacks?”.

The respondents had the following options to respond according to their breakfast consumption:

“I never have breakfast on weekdays”, “1 day”, “2 days”, “3 days”, “4 days”, “5 days”.

The response categories for snack consumption were “only midmorning”, “only afternoon”, “both midmorning and afternoon”. Each independent variable was dichotomized into regular and irregular meal intake. Breakfast consumed on “3 weekdays or less” and not having snacks both “midmorning” and “afternoon” was considered as irregular behaviour, even if the IGHD advise is for having breakfast and two snacks every day [32].

#### Covariates

Several factors related to skipping meals and fruit and vegetable consumption among adolescents were established. These factors were defined as covariates and included age, sex, socioeconomic status, family type, body perception, weight status and intended weight loss [33-37]. Due to the small age variation in the sample, school grades were used as a substitute for age, specifically grades 11, 13 and 15. The “social class” (socioeconomic status) was based on the Family Affluence Scale (FAS). FAS is used as a measure of socioeconomic status. The scale’s characteristics as well as its modality of use has been reported by Currie et al. [28]. The scale consists of four items: number of family-owned cars (no = 0, one = 1, two or more = 3), having one’s own bedroom (no = 0, yes = 1), number of family holidays last year (none = 0, once = 1, twice and more = 2) and number of family-owned computers (none = 0, one = 1, two and more = 2). A composite FAS score was calculated by summing up the points assigned to each response. The score was converted into a three-point ordinal scale where a FAS score of 0–3 was categorized as low affluence, a score of 4–5 as medium affluence and a score of 6–7 as high affluence. The variable family type was constructed from the respondents’ reports of who they lived with. Respondents were categorized into four categories “Traditional family” (living with two biological parents), “Single parent”, “Reconstructed family” (Living with one parent and one step-parent) and “Other family types” (e.g. foster homes).

As for the body perception, respondents were asked to complete the phrase: “Do you think your body is…”, with any of the following options: “Much too thin”, “Too thin”, “More or less the right size”, “Too fat” and “Much too fat”. The variable was dichotomized by grouping the respondents who answered, “A bit too fat” and “Much too fat” together, along with residual categories.

The respondents were asked to declare their weight without clothes and their height without shoes. Weight status (dichotomized into overweight/not overweight) was defined by using the BMI cut-off points set by the International Obesity Task Force [38]. Self-reported weight and height has limitations as it often leads to a BMI underestimation [39]. The “intended weight loss” was measured through the following two questions: “How often have you dieted with the aim of losing weight during the last 12 months?” and “Are you on a diet, or do you do other things to lose weight?”. The options for the first question were the following: “never”, “1-2 times”, “3-4 times”, “5-6 times” and “7 times or more”. The options for the second question were: “No, because I have to gain weight”, “No, but I ought to lose weight”, “No, my weight is fine” and “Yes, I am trying to lose weight”. The variable was dichotomized into participants who were trying to lose weight and those who were not.

#### Statistical analyses

All the analyses were carried out using SPSS 20.0 (SPSS Inc., Chicago, IL, USA) statistical software package. All the categorical variables are expressed with number of cases (%), and the continuous variables with mean (sd). In the descriptive analysis, we tested sex and age differences by Chi2 test and used Cochran’s test to test for trend. Multivariate logistic regression analyses were used to study the association between meal intake (breakfast and snacks) and fruit and vegetable consumption. Initially, we analyzed the entire sample implementing two models: one taking into account the consumption of fruit as the dependent variable and the other taking into account the consumption of vegetables, always as the dependent variable. The covariates included in both models were breakfast and snack consumption. The models were adjusted for age, sex, socioeconomic status, family type, body perception, weight status and intended weight loss. In the next step the analyses were stratified by sex to assess whether sex was an effect modifier (moderator). To evaluate the modifying effect of age, the final analyses were also stratified by age. Additionally, the modifying effect of sex and age was tested by including an interaction term in the statistical models. Throughout the paper, statistical significance has been defined by conventional levels of p < 0.05.

## Results

### Descriptive analysis

Table 1 shows the participant’s distribution by each variable. The adolescents included in the HBSC study were aged 11, 13 and 15 years. Data was obtained from a total sample of 3291 students of which 1135 were 11-year-olds (34.5%), 1255 were 13-year-olds (38.1%) and 901 were 15-year-olds (27.4%), with a gender mix of 1702 males (51.7%) and 1589 females (48.3%). The mean ages of the respondents aged 11, 13 and 15 years were respectively 11.41, 13.43 and 15.54 years. Boys eat significantly less fruit than girls. Boys who were classified as low fruit consumers constituted 61.6% of the sample compared with 56.6% for girls (χ2 = 8.35; p = 0.004). Similarly, when considering vegetable consumption, boys reported a lower consumption frequency than girls, with 75.5% of boys classified as low consumers compared with 66.2% of girls (χ2 = 34.46; p < 0.0001). Breakfast was more frequently skipped by girls (35.1%) as compared with boys (26.2%) (χ2 = 30.24; p < 0.0001), whereas boys tended to skip snacks more often than girls (50.0% vs. 41.8% respectively; χ2 = 22.17; p < 0.0001). The proportion of boys with a low frequency of fruit intake increased with age (χ2 for trend = 22.08; p < 0.0001) like irregular breakfast habits (χ2 for trend = 34.31; p < 0.0001). For low fruit intake and irregular breakfast and snack consumption, the stepwise age gradient was a statistically significant result (χ2 = 23.85, p < 0.0001; χ2 = 34.52, p < 0.0001; χ2 = 10.69, p = 0.005 respectively). For low vegetable intake, no meaningful significant results were registered (data is not shown). Compared with 11-year-olds, 15-year-olds face nearly twice the risk of irregular fruit intake (OR15y vs. 11y = 1.87 (1.45-2.42)). The risk of irregular breakfast intake is higher for 13-year-olds (OR13y vs. 11y = 1.67 (1.27-2.19)) and 15-year-olds (OR15y vs. 11y = 2.32 (1.74-3.08)). In the context of irregular snack intake, there emerged a reverse association with age, since 13-year-olds run a lower risk of consuming irregular snacks than 11-year-olds (OR13y vs. 11y =0.69 (0.55-0.86)) (data is not shown). The proportion of girls with a low frequency of fruit intake increased with age (χ2 for trend = 4.80; p = 0.03) like irregular breakfast habits (χ2 for trend = 47.29; p < 0.0001) and irregular snack habits (χ2 for trend = 11.87; p = 0.001) (data is not shown). For irregular breakfast and snack consumption, the stepwise age gradient was a statistically significant result (χ2 = 48.12; p < 0.0001; χ2 = 15.08; p = 0.001 respectively) (data is not shown). Compared with 11-year-olds, 15-year-olds face a 33% higher risk of irregular fruit intake (OR15y vs. 11y = 1.33 (1.03-1.72)). Regarding irregular breakfast intake, 13-year-olds (OR13y vs. 11y = 1.83 (1.42-2.37)) and 15-year-olds (OR15y vs. 11y = 2.59 (1.96-3.40)) face a higher risk when compared with 11-years-olds. When looking at irregular snack intake, we can observe a direct association with age, since 13-year-olds run a higher risk to consume irregular snacks than 11-year-olds (OR15y vs. 11y = 1.59 (1.23-2.05)) (data is not shown).

**Table 1:** The schoolchildren described by the applied variables, % (n)

	**Boys**						**Girls**						**Total**
	**11 years**		**13 years**		**15 years**		**11 years**		**13 years**		**15 years**		
	**%**	**N**	**%**	**N**	**%**	**N**	**%**	**N**	**%**	**N**	**%**	**N**	**N = 3291**
**Dependent variables**													
***Low frequency of fruit intake***													
*Yes*	56.0	335	60.1	382	70.3	329	53.1	285	56.7	351	60.3	261	1943
*No*	43.6	261	39.8	253	29.3	137	46.6	250	43.1	267	39.7	172	1340
*Missing*	0.4	2	0.1	1	0.4	2	0.3	2	0.2	1	0.0	0	8
***Low frequency of vegetable intake***													
*Yes*	75.1	449	72.5	461	78.2	366	65.0	349	68.2	422	64.0	277	2324
*No*	23.9	143	27.0	172	21.4	100	34.3	184	31.7	196	35.8	155	950
*Missing*	1.0	6	0.5	3	0.4	2	0.7	4	0.1	1	0.2	1	17
**Independent variables**													
***Breakfast consumption***													
*Irregular*	17.9	107	27.0	172	34.0	159	24.0	129	36.8	228	45.3	196	991
*Regular*	79.3	474	71.9	457	65.0	304	74.5	400	62.4	386	54.3	235	2256
*Missing*	2.8	17	1.1	7	1.0	5	1.5	8	0.8	5	0.4	2	44
***Sneak consumption***													
*Irregular*	53.3	319	45.4	289	50.0	234	37.8	203	39.4	244	49.7	215	1504
*Regular*	44.0	263	54.6	347	49.6	232	60.9	327	60.6	375	50.3	218	1762
*Missing*	2.7	16	0.0	0	0.4	2	1.3	7	0.0	0	0.0	0	25
**Covariates**													
***Nutritional status***													
*Overweight*	15.1	90	14.0	89	14.1	66	10.1	54	9.0	56	9.2	40	395
*Not overweight*	65.2	372	58.3	371	75.6	354	57.9	311	76.9	476	83.4	361	2245
*Missing*	22.7	136	27.7	176	10.3	48	32.0	172	14.1	87	7.4	32	651
***Intended weight loss***													
*Yes*	13.2	79	10.7	68	8.5	40	12.3	66	19.1	118	23.8	103	474
*No*	86.3	516	88.7	564	91.5	428	87.5	470	80.8	500	76.0	329	2807
*Missing*	0.5	3	0.6	4	0.0	0	0.2	1	0.1	1	0.2	1	10
***See oneself as fat***													
*Yes*	19.9	119	19.3	123	17.3	81	22.2	119	27.0	167	33.0	143	752
*No*	78.6	470	79.6	506	82.3	385	77.7	417	72.5	449	66.1	286	2513
*Missing*	1.5	9	1.1	7	0.4	2	0.1	1	0.5	3	0.9	4	26
***Family type***													
*Traditional*	76.6	458	79.1	503	80.3	376	79.1	425	79.5	792	79.0	342	2596
*Single parent*	13.4	80	14.3	91	16.5	77	15.1	81	16.6	103	18.7	81	513
*Others*	3.7	22	2.0	13	1.3	6	1.1	6	1.3	8	1.4	6	61
*Missing*	6.3	38	4.6	29	1.9	9	4.7	25	2.6	16	0.9	4	121
***FAS***													
*High*	49.0	293	55.7	354	58.1	272	42.6	229	53.2	329	49.0	212	1689
*Medium*	36.0	215	36.3	231	34.8	163	44.9	241	40.2	249	41.6	180	1279
*Low*	9.4	56	6.0	38	5.8	27	10.8	58	5.8	36	7.9	34	249
*Missing*	5.6	34	2.0	13	1.3	6	1.7	9	0.8	5	1.5	7	74

A much higher number of girls than boys (18.1% vs. 11.0%, χ2 = 33.69; p < 0.0001) reported that they were trying to lose weight. 27.1% of girls perceived themselves as fat compared with 19.2% of boys (χ2 = 28.67; p < 0.0001) (data is not shown).

### Multivariate analysis

#### Breakfast consumption and fruit intake

As illustrated in Table 2, a low frequency of fruit intake was associated with irregular breakfast consumption (OR = 1.28 (1.07-1.53)). When the analysis was stratified by sex, a significant association was found among girls (OR = 1.31 (1.02-1.67)), but not among boys (OR = 1.25 (0.96-1.64)). Among 15-year-old girls, the association between irregular breakfast habits and fruit intake was a statistically significant result (OR = 1.52 (1.01-2.31)).

**Table 2:** Adjusted OR (CI 95%) for low fruit intake by breakfast and snacks frequency

	**All school children**	**Boys**	**Girls**	**Sex interaction**	**Boys**			**Age-interaction**	**Girls**			**Age-interaction**
				**P-value**	**11 years**	**13 years**	**15 years**	**P-value**	**11 years**	**13 years**	**15 years**	**P-value**
**Breakfast**												
*Irregular vs. Regular*	**1.28 (1.07–1.53)**	1.25 (0.96–1.64)	**1.31 (1.02–1.67)**	0.79	1.51 (0.88–2.58)	1.30 (0.84–2.00)	1.09 (0.67–1.75)	0.75	1.25 (0.73–2.15)	1.14 (0.78–1.66)	**1.52 (1.01–2.31)**	0.73
**Snacks**												
*Irregular vs. Regular*	**1.20 (1.02–1.42)**	1.25 (0.99–1.59)	1.18 (0.94–1.49)	0.76	**1.73 (1.16–2.59)**	1.01 (0.69–1.50)	1.03 (0.66–1.61)	0.12	1.41 (0.90–2.23)	**1.45 (1.01–2.09)**	0.75 (0.49–1.14)	**0.04**

More covariates were applied for the purposes of the full population research, such as: age, body perception, gender, family social class and type and intended weight loss.

The analyses that were stratified by sex were adjusted based on the same covariates except sex.

The ones that were stratified by age and sex were adjusted based on the same covariates except sex and age.

Bold: significant OR.

No statistically significant interaction was found between sex and age.

#### Snack consumption and fruit intake

Significant association between irregular snack consumption and low frequency of fruit intake was found (OR = 1.20 (1.02-1.42)). No sex interaction was found, while age interaction was found only among girls (p = 0.04). As shown in Table 2, the OR was significant for 11-year-old boys (OR = 1.73 (1.16-2.59)) and 13-year-old girls (OR = 1.45 (1.01-2.09)).

#### Breakfast consumption and vegetable intake

A significant association was found between irregular breakfast intake and low frequency of vegetable consumption (OR = 1.31 (1.07-1.60)). No sex and age interactions were found. The girls with irregular breakfast consumption had a significant risk (OR = 1.40 (1.07-1.82)) of a low vegetables intake as well. As shown in Table 3, the only significant association that emerged was for 15-year-old girls (OR = 1.54 (1.01-2.38)).

**Table 3:** Adjusted OR (CI 95%) for low vegetable consumption by breakfast and snacks frequency

	**All school children**	**Boys**	**Girls**	**Sex interaction**	**Boys**			**Age-interaction**	**Girls**			**Age-interaction**
				**P-value**	**11 years**	**13 years**	**15 years**	**P-value**	**11 years**	**13 years**	**15 years**	**P-value**
**Breakfast**												
*Irregular vs. Regular*	**1.31 (1.07–1.60)**	1.22 (0.89–1.66)	**1.40 (1.07–1.82)**	0.55	0.88 (0.48–1.60)	1.55 (0.93–1.57)	1.14 (0.66–1.97)	0.38	0.99 (0.55–1.76)	1.49 (0.98–2.67)	**1.54 (1.01–2.38)**	0.46
**Snacks**												
*Irregular vs. Regular*	1.03 (0.86–1.24)	1.08 (0.83–1.42)	1.00 (0.78–1.28)	0.65	0.93 (0.58–1.48)	0.88 (0.56–1.37)	**1.91 (1.13–3.25)**	0.13	1.50 (0.92–2.47)	1.12 (0.75–1.68)	**0.63 (0.41–0.97)**	**0.04**

Study on full population; covariates: age, intended weight loss, sex, body perception and family type and social class.

The analyses that were stratified by sex were adjusted based on the same covariates except sex.

The ones that were stratified by age and sex were adjusted based on the same covariates except sex and age.

Bold: significant OR.

#### Snack intake and vegetable consumption

As seen in Table 3, the results do not indicate a meaningful association between irregular snack consumption and low frequency of vegetable intake.

No sex interaction was found, but age interaction was found only among girls (p = 0.04).

The only significant association was found for 15-year-old boys and girls. For the boys, irregular snack consumption is a risk factor (OR = 1.91 (1.13-3.25)), while for the girls it is a protective factor (OR = 0.63 (0.41-0.97)).

## Discussion

The aim of this research was to observe whether there were any direct associations between the frequency of breakfast and snack consumption and fruit and vegetable intake among 11-, 13- and 15-year-old Tuscan adolescents. Association was confirmed between irregular breakfast habits and low frequency of fruit and vegetable consumption, and between irregular snack consumption and low frequency of fruit intake. No association was found between irregular snack consumption and low frequency of vegetable consumption.

In the analyses stratified by gender, the relation between irregular breakfast habits and fruit and vegetable consumption emerged only for the female respondents, and they appeared to be statistically consistent.

Different trends appeared when the sample was stratified by age and sex. Overall, irregular breakfast habits represented a lower risk factor for low frequency of fruit and vegetable consumption among younger girls compared with their older counterparts. An opposite pattern emerged when we considered irregular snack consumption among girls. It is important to note that the age trend changed between boys and girls and depended on whether it was fruit or vegetable intake that was considered. The association between fruit and vegetable consumption and meal frequency among teenagers is supported by other research in the field [15-19], even though the methods and instruments used to measure meal frequency and fruit and vegetable intake were different. The modifying effect of sex has not been examined in depth by any study so far.

Melnik et al. [40] carried out a study which showed how American children who missed meals had a lower fruit and vegetable intake, across all age groups that were considered. However, in contrast, the current study on fruit and vegetable intake and irregular eating habits has yielded several different findings for different age groups. There are many reasons for this difference in findings between the two studies. First, the analysis by Melnik et al. collected data by using a combined scale of fruit and vegetable consumption. Second, the age groups were younger than those considered in the present study. Third, their study did not stratify the analysis by gender.

There is only one similar study that has considered the modifying effects of age and gender [41]. In that study, contrary to the results reported in the present study, it emerged that skipping breakfast, particularly among girls, represented a less serious risk of lower fruit and vegetable intake in younger school children. A possible explanation for this divergence in results could be that younger children have more opportunities to consume fruits and vegetables even if they skip meals, as parents have more control over their diet. Older children, on the other hand, are more independent and their family’s influence on their eating habits is less [42,43].

Breakfast seems to be a determinant of vegetable intake. A strong association between irregular breakfast and low frequency of vegetable consumption was found for 15-year-old girls. This could indicate, more generally, that skipping breakfast is an indicator of unhealthy eating habits in this population. This explanation would be in consonance with other studies, which have shown that irregular breakfast habits are associated with poor nutrition [15,18,37]. The results of this study did not permit generalizations for irregular snack intake because the pattern consistently varied between gender, age groups and fruit and vegetable intake.

### Strengths and limitations

The representativeness of the sample studied and the implementation of tested and validated methods and tools as documented in various studies [22] represent a strength of the present study.

As shown in literature, the validity of self-reported dietary assessment procedures among teenagers yields diverging results [29]. FFQ is recognized as a valid tool for ranking teenagers based on their habitual food and beverage consumption [29,44,45].

A HBSC study from 2004/05 validated the question on breakfast frequency. It showed a reasonable accordance with food habits reported in diaries (kappa statistics 0.47) [46].

A further limitation of the study may be associated with the fact that only weekdays were considered for measuring frequency. We considered only weekdays because eating routines might be different during weekends. Studies have shown that generally people consume more caloric foods and generally larger portions during the weekend [47-49]. The family’s overall nutritional and meal consumption attitude could represent an underlying unmeasured confounding factor. A positive connection between family attitude towards meal consumption and children’s consumption of fruit, vegetables and breakfast has been reported in other studies [33,34,42]. The association between meals consumed within the family and fruit and vegetable intake may indicate an overall positive family (home) food environment where the availability of fruits and vegetables is higher and healthy meal habits are being supported [43,49].

## Conclusions

The findings of this study suggest that among 11-,13-and 15-year-old school children, irregular meal consumption is associated with low fruit and vegetable consumption.

From a stratified analysis, this association proved stronger among 15-year-olds and in particular among female students. The importance of using separate measures to estimate the consumption of fruits, vegetables, breakfast and snacks is indicated by different patterns of consumption that emerged in this study. The findings of the present research underline the importance of promoting regular meal intake as part of a healthy nutritional routine among teenagers, as well as encouraging the consumption of fruits and vegetables. It also emerged from this study that health promotion campaigns that aim to promote regular meal consumption and increase consumption of fruits and vegetables need to take into account gender and age differences in designing promotion strategies.

The majority of teenager meals is consumed in family and school environments. To ameliorate eating and nutritional habits in the family environment, it is suggested to involve the parents of school-aged children in health-promotion interventions. This emphasizes the value of healthy eating habits and gives parents the tools to establish a healthy eating routine [9,16,34,42,50].

In the school environment, the promotion of healthy eating habits should aim at increasing the quality and availability of meals in schools. Knowledge and skill training are needed to improve food consumption patterns. Furthermore, promoting healthy eating habits and increasing the amount of fruits and vegetables consumed by children is not an objective in itself; studies have demonstrated that breakfast and lunch programmes in schools lead to secondary health benefits as a result of higher fruit and vegetable consumption [40,51]. Factors that may motivate young people to consume more fruits and vegetables include a change in the environment by, for example, increasing the availability of fruits and vegetables at home and promoting parental consumption [33], providing fruits and vegetables in schools [52] and implementing a schoolyard garden with appropriate educational activities [53]. Teachers and health professionals can also help through targeted school interventions, which have consistently been shown to increase intake [33].

Our study provides current and detailed information regarding fruit, vegetable, breakfast and snack consumption habits and compliance with the IGHD recommendations, which can inform policymakers and health promoters about the need for intervention to improve alimentary behaviour. Despite the possible increase in overall breakfast frequency, health promotion efforts should still aim at stimulating daily breakfast consumption among specific subgroups identified in our study. So far, there is no clear overview of intervention programmes to stimulate breakfast consumption in the Italy. It is our impression that most initiatives are regional, often aimed at lower age groups, and they do not affect evaluations. Further research needs to examine whether more stringent adherence to the IGHD recommendations will result in enhanced fruit and vegetable intake at breakfast and as a snack. Additional research is also necessary to further assess personal and environmental determinants of breakfast and snack behaviour.

## Abbreviations

FFQ: Food frequency questionnaire; BMI: Body Mass Index; FAS: Family affluence scale; IGHD: Italian guidelines for a healthy diet.

## Competing interests

The authors declare that they have no competing interests.

## Authors’ contributions

GL wrote the manuscript and organized the data collection; EA collaborated in interpreting the data and the preparation of the manuscript; AP performed statistical analyses; RS collaborated in organizing the data collection and input; VM collaborated in the preparation of the manuscript; DdW contributed in writing the manuscript as well as the final review thereof; MVG collaborated in the outline of the study, and the final review of the manuscript. All authors have read and approved the final manuscript.
